# Spontaneous recurrent menstrual pneumothorax: a case report

**DOI:** 10.1097/MS9.0000000000001592

**Published:** 2023-12-05

**Authors:** Takae Hirono, Ye Feng, Wenhui Wang, Huan Yu

**Affiliations:** a Peking University Health Science Center; b Department of Obstetrics and Gynaecology, China-Japan Friendship Hospital, Beijing, China; c The University of Warwick, Coventry, UK

**Keywords:** case report, catamenial pneumothorax, dienogest, oral contraceptives, thoracic endometriosis, VATS

## Abstract

**Introduction and importance:**

Endometriosis is most commonly found in the pelvic area, ~12% of people have it in other areas or organs, which is known as extrapelvic endometriosis. Thoracic endometriosis, which is also classified as extrapelvic endometriosis, manifests with four distinct forms: catamenial pneumothorax, catamenial hemothorax, catamenial hemoptysis, or lung nodules. Catamenial pneumothorax is the most common clinical symptom of these; however, it is frequently neglected by clinicians and goes undiagnosed and untreated. As a result, it is critical to raise awareness of this medical condition among clinicians.

**Case presentation:**

The authors present a case report of a 34-year-old woman of reproductive age who had recurrent episodes of spontaneous pneumothorax during menstruation and underwent treatment with thoracoscopic surgery as well as gynaecological hormonal drugs including oral progesterone and dienogest throughout this time. Based on her symptoms, a catamenial pneumothorax caused by thoracic endometriosis was suspected.

**Clinical discussion:**

The clinical symptoms, pathogenesis, diagnosis, and treatment of Catamenial Pneumothorax are analyzed. Furthermore, the usage of gynaecological hormone medications in this condition has been discussed. The mechanisms of oral contraceptives and progestin-based medications are evaluated by comparing the patient’s treatment process, highlighting their pros and cons.

**Conclusions:**

Thoracoscopic surgery combined with postoperative gynaecological hormonal medications may be the most effective treatment for this issue. Several gynaecological hormonal medicines are available, each of which has its own set of pros and cons, and must be thoroughly evaluated as well as correctly tailored to the patient’s specific circumstances to have a positive therapeutic outcome.

## Introduction

Highlights

- This case reports a clinically unusual condition, catamenial pneumothorax, which requires both gynaecology and thoracic surgery, and might be utilized for physicians’ reference and knowledge.
- In terms of diagnosis and treatment, two distinct modules are split into thoracic surgery and gynaecology, underlining the significance of integrated treatment of both.
- The mechanism of dienogest’s usefulness in the treatment of catamenial pneumothorax and even endometriosis is specifically described.

Catamenial pneumothorax (CPTX) refers to spontaneous and recurrent pneumothorax occurring in reproductive-age females during their menstrual period. Most cases manifest within 72 h after the onset of menstruation, displaying symptoms such as chest pain, breathlessness, and cough. Catamenial Pneumothorax is observed in over 90% of cases on the right side, less frequently on the left side, and bilateral occurrences are rare^1,2^. It is closely associated with thoracic endometriosis syndrome (TES), including manifestations like menstrual hemothorax, hemoptysis during menstruation, and pulmonary nodules, all attributed to thoracic endometriosis^3^. Some cases of Catamenial Pneumothorax might be non-TES related, hypothesized to result from elevated levels of prostaglandin F2-alpha during the menstrual cycle, causing bronchial and vascular constriction, leading to alveolar rupture and subsequent bulla formation^4,5^.

The exact pathogenesis of thoracic endometriosis syndrome remains inconclusive, attributed to the combined effect of multiple factors, with retrograde menstruation theory being a prominent hypothesis. This theory suggests that functional endometrial tissue retrograde into the pelvic cavity through the fallopian tubes, implants on the ovaries and pelvic peritoneum, forming ectopic foci. Subsequently, endometrial cells within the pelvic cavity are carried by peritoneal fluid, which flows from the pelvis to the right hemidiaphragm via the right paracolonic sulcus but is deflected from the left hemidiaphragm by the obstruction of the falciform and phrenicocolic ligaments, so that it mainly remains in the right suprahepatic space^3^. Upon reaching the region below the right diaphragm, implants occur on the diaphragmatic surface or pass through diaphragmatic fenestrations to invade and damage pleura and lung parenchyma, culminating in pneumothorax-like symptoms. This theory also accounts for the preponderance of right-sided occurrences in Catamenial Pneumothorax^3,6,7^. An alternative benign metastasis theory posits that endometrial cells can disseminate via lymphatics and blood vessels to distant organs, forming ectopic lesions^3,8^. Supporting evidence includes identifying circulating endometrial cells in the peripheral blood of patients with endometriosis^9^, and reports of endometrial lesions in distant organs such as the brain, bones, and eyes^3,8^.

## Clinical data

**Figure 1:**
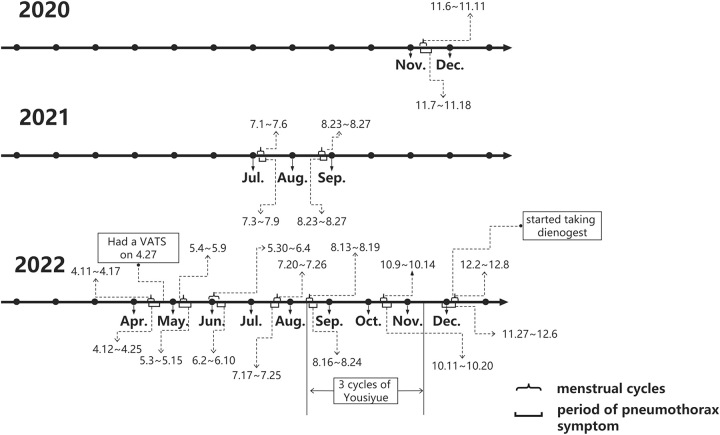
The graph illustrates the temporal correlation between the onset of pneumothorax symptoms, as per the patient’s self-recorded schedule, and the concurrent menstruation episodes spanning from 2020 to 2022.

**Figure 2:**
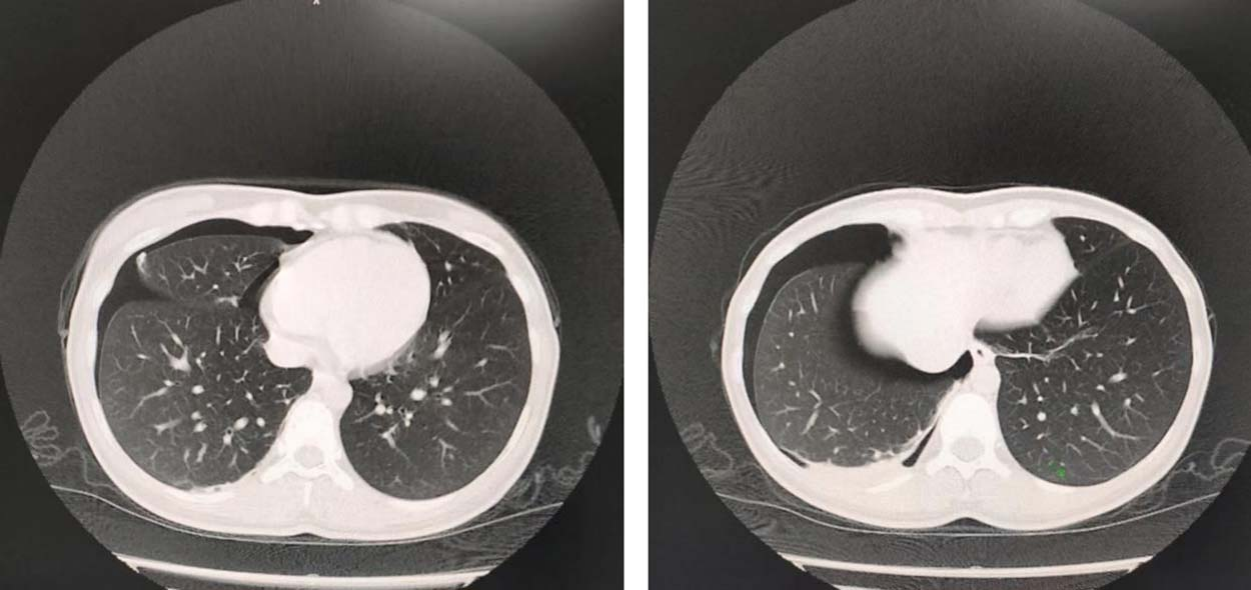
Computed tomography images taken during the patient’s first recurrence after surgery, revealing compressed lung tissue.

**Figure 3:**
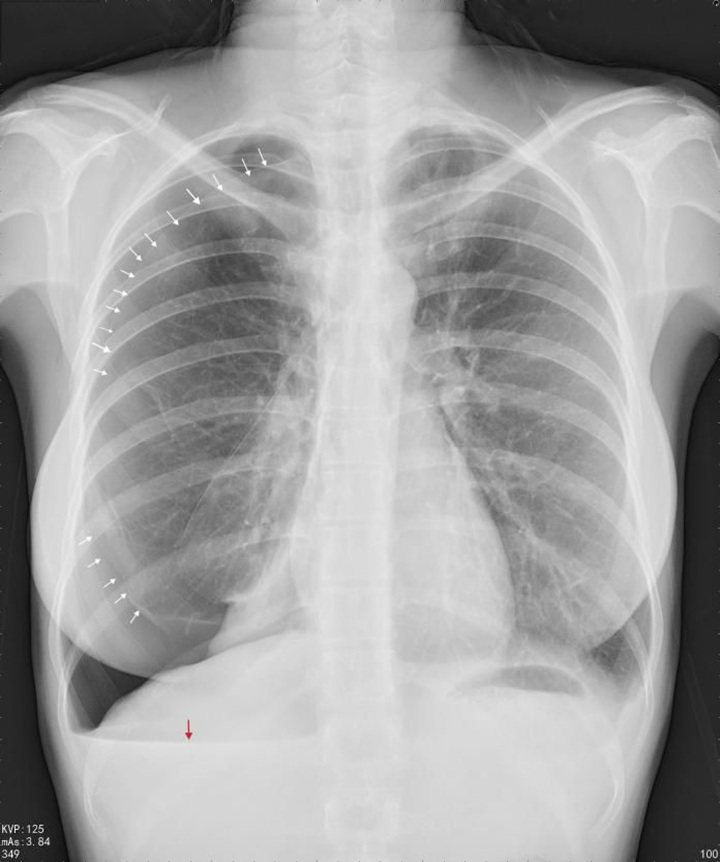
Chest radiograph captured during the patient’s first recurrence after surgery, with the white arrow indicating the visceral pleural line and the red arrow indicating the air-fluid level.

The patient, a 34-year-old woman with a history of good health, regular menstruation, and dysmenorrhoea, unmarried and nulliparous (G0P0), began experiencing chest pain and breathlessness without apparent triggers during her menstrual period since 2020. There were three similar episodes between 2020 and 2021. The relationship with her menstrual cycle is shown in Figure 1. No examinations or treatments were undertaken during these incidents. Symptoms improved after the menstrual period, taking around 10 days for full recovery. On 12 April 2022, she had sudden chest pain and breathlessness during her menstrual period and underwent a chest computed tomography (CT) examination at an external hospital. The CT scan indicated right-sided pneumothorax with 20% compression of the right lung. On 27 April, the patient underwent video-assisted thoracoscopic surgery (VATS) to resect a bulla on the right upper lobe, performed by an attending surgeon in the Department of Thoracic Surgery at our institution. Intraoperatively, a bulla was found at the apex of the right upper lobe. Postoperative pathology confirmed a bulla in the upper lobe of the right lung. However, the postoperative treatment outcomes were unsatisfactory, with recurring symptoms from May to August, verified by lateral chest radiographs as recurrent right-sided pneumothorax (Figs 2, 3). Conservative observation was selected. Due to findings of uterine fibroids and endometrial polyps on transvaginal pelvic ultrasound, she started a three-month regimen of Yousiyue (1 tablet daily), taken between August and November, which is the trade name of drospirenone and ethinylestradiol tablets, a new type of oral contraceptive pill, consisting of ethinylestradiol 0.02 mg and drospirenone 3.0 mg. Yet another episode occurred in October and the Continuous conservative observation was adopted. Since December 2022, she has started taking Dienogest (2 mg/day). One episode occurred during the medication-free period in April 2023; however, she remained under conservative observation. The patient experienced no adverse reactions from the treatment, but due to the need to prepare for pregnancy, the patient will consider discontinuing the prescription.

This case has been reported in line with the SCARE criteria^10^.

## Discussion

Based on the clinical data presented above, a preliminary diagnosis of Catamenial Pneumothorax can be inferred. The diagnosis of Catamenial Pneumothorax primarily hinges on the patient’s medical history and symptoms. Reproductive-age females presenting with recurrent chest pain, dyspnoea, and cough during the perimenstrual period, alleviating after menstruation cessation, suggest Catamenial Pneumothorax^7^. Complementary imaging techniques aid in diagnosis, with X-rays and CT scans revealing pneumothorax, pleural effusion, and, in some cases, diaphragmatic defects and other thoracic endometriosis lesions visible via CT and MRI^3,6^. Subsequent definitive diagnosis and treatment methods are closely intertwined. Therefore, the following discussion will encompass both diagnostic and therapeutic approaches.

Catamenial Pneumothorax entails diagnostic and therapeutic aspects from both thoracic surgery and gynaecology:

Thoracic surgery: Thoracoscopic surgery plays a pivotal role in diagnosing and treating Catamenial Pneumothorax and even thoracic endometriosis. Regarding diagnosis, thoracoscopy not only enables direct visualization of lesions but also allows for the acquisition of pathological specimens for tissue analysis, thereby confirming the diagnosis of Catamenial Pneumothorax caused by thoracic endometriosis^11^. Under thoracoscopy, characteristic pathological findings in Catamenial Pneumothorax patients include multiple minor defects in the diaphragm. Tissue samples obtained from the edges of these defects often reveal endometrial tissue^3,8,11^. Additionally, punctate or nodular lesions may be observed, varying in colours like red, brown, or purple. These lesions can appear on the diaphragm, visceral pleura, parietal pleura, and lung parenchyma, likely representing endometrial implants, as histological examination often detects endometrial tissue^8^. Some patients may exhibit atypical changes during surgery, with only the presence of lung bullae^8,11^.

Treatment: Employing thoracic surgery can effectively reduce the recurrence rate of Catamenial Pneumothorax, surpassing the use of gynaecological drugs alone^8,11,12^. In a retrospective study by Jos Joseph and colleagues involving 80 Catamenial Pneumothorax patients, it was found that the recurrence rate in the hormone treatment group was notably higher than that in the surgical treatment group. Even after 6 months of hormonal maintenance therapy, the recurrence rate was already around 50%^12^. Successful treatment of thoracic endometriosis necessitates eradication or inhibition of existing endometriotic lesions. Relying solely on hormonal treatment is not entirely effective in regressing endometrial implants. Hence, surgical intervention is required to remove visible lesions^3,5,8,11,12^. Since migrating endometrial cells to the thoracic cavity is a continuous process, meticulous handling of the diaphragm during surgery is crucial in treating Catamenial Pneumothorax. The optimal approach involves resecting the affected diaphragm rather than simply suturing the defect^13^. Suturing alone may leave endometrial implants in place, potentially leading to recurrent diaphragmatic perforation post-surgery and subsequent dispersion of endometrial cells into the thoracic cavity^13,14^. The presentation of this condition within the thoracic cavity exhibits considerable individual variation; hence, specific surgical techniques should be tailored to the patient. It is advisable to perform surgery during the menstrual period to observe the lesions better^8,11^.

Gynaecological treatment: Postoperative use of gynaecological hormone medications effectively alleviates symptoms and reduces recurrence. However, no universally accepted optimal medication regimen for this condition exists. Therefore, in the current stage, especially for cases of Catamenial Pneumothorax where thoracic endometriosis is definitively diagnosed, postoperative medication approaches are generally similar to those used for pelvic endometriosis^6^. Additionally, a substantial portion of patients with thoracic endometriosis have been verified to have pelvic endometriosis^3,6,8^. Hormonal drugs used to treat endometriosis mainly include combined oral contraceptives, progestins, gonadotropin-releasing hormone (GnRH) agonists, and GnRH antagonists^15^. Among these, GnRH agonists are believed to be more effective than hormone medications that preserve menstruation in controlling Catamenial Pneumothorax. However, due to their significant side effects, their treatment duration should not exceed 6 months^5,13,16,17^. Similarly, other hormonal medications can also be used for postoperative treatment of Catamenial Pneumothorax, but there is a lack of prospective cohort studies to analyze the comparative efficacy^11,18^.

In this case, a combined oral contraceptive, Yousiyue, was initially administered, containing ethinylestradiol and drospirenone. However, symptoms recurred in the third month of usage. Later, Dienogest was prescribed. This highly effective progestin drug has garnered attention in recent years. It inhibits ovulation through central and peripheral mechanisms. Centrally, it moderately suppresses the secretion of follicle-stimulating hormone and luteinizing hormone in the hypothalamic-pituitary-ovarian axis, thereby inhibiting follicular development and ovulation. As it permits the development of sinusoidal follicles, it maintains early follicular phase oestrogen levels (30–50 pg/ml), thereby reducing the risk of menopausal symptoms and bone loss. Peripherally, it inhibits aromatase, cyclooxygenase, and prostaglandin E2 synthesis, reducing oestrogen levels and promoting apoptosis of dominant follicular granulosa cells to inhibit ovulation^19^. Additionally, Dienogest binds to progesterone receptors and exhibits local anti-proliferative, anti-inflammatory, and anti-angiogenic effects on endometriosis lesions, distinguishing it from other progestins^13^.

Currently, both combined oral contraceptives and progestins are considered first-line treatments for endometriosis^15,20^. Prospective cohort studies by Ilaria Piacenti and Lina El Taha have shown that both Dienogest and combined oral contraceptives effectively relieve endometriosis-related pain, including non-cyclic pelvic pain, dysmenorrhoea, and dyspareunia, improving patients’ quality of life^21,22^.

However, there are cases like the one presented here where combined oral contraceptives prove ineffective, and in recent years, there have been some questioning voices regarding their use^20,23–25^. Studies by Ilaria Piacenti and Stefano Angioni have shown that combined oral contraceptives for six months only minimally reduce the size of endometriotic lesions, whereas Dienogest effectively reduces lesion volume^21,26^. Low-dose combined oral contraceptives contain 20–30 mg of ethinylestradiol (EE), which, when converted, is equivalent to 4–6 times the physiological dose of oestrogen^20,23,24^. Simultaneously, basic research has confirmed that due to alterations in oestrogen receptors (ER) and progesterone receptors (PR) in ectopic endometrial cells compared to eutopic endometrial cells, they do not respond adequately to progesterone, which means there is a certain progesterone resistance in ectopic endometrium^23^. In this scenario, oestrogenic components in oral contraceptives might paradoxically dominate in the ectopic lesions, stimulating lesion growth and potentially increasing the risk of progressing into deep infiltrating endometriosis (DIE). Some suggest that past use of oral contraceptives for primary dysmenorrhoea and endometriosis, particularly DIE, might be interrelated^20,23,24,26^.

Dienogest’s efficacy under progesterone resistance can be attributed to its mechanisms beyond ovulation suppression only. Its local endocrine environment is characterized by low oestrogen and high progesterone, which induces decidualization and subsequent atrophy of endometrial tissue^27^. Its local anti-proliferative, anti-inflammatory, and anti-angiogenic effects are deemed essential^21,27,28^. Dienogest inhibits proliferation in endometrial-like tissue directly and does not rely on progesterone receptors. Both in vitro and in vivo experiments have confirmed its indirect anti-inflammatory activity by modifying pro-inflammatory factors^27^. Dienogest also regulates vascular growth in ectopic endometrial lesions by suppressing vascular growth factors, leading to reduced microvascular network size, lowered microvessel density, and subsequent vessel dilation^25^. All these suppressed processes are crucial aspects of endometriosis development, indicating that Dienogest achieves lesion regression through multiple mechanisms.

## Conclusion

Catamenial Pneumothorax is relatively uncommon in clinical practice, which can lead to it being overlooked or misdiagnosed. It is essential to raise awareness about this condition among thoracic and gynaecology medical professionals to ensure timely and accurate diagnosis and treatment for patients. In the case presented, the patient’s recurrent episodes associated with her menstrual history were not adequately considered. Consequently, she underwent only a pleural resection surgery in the thoracic department. Residual endometrial lesions will likely remain within the thoracic cavity, as evidenced by her subsequent recurrent episodes. In persistent lesions, Yousiyue’s medication was ineffective in controlling the condition. This may be attributed to the previously mentioned theory of progesterone resistance and the stimulative effect of oestrogen components found in oral contraceptives, preventing lesion reduction. In contrast, Dienogest effectively controlled the patient’s lesions, possibly due to its ability to induce lesion regression through multiple mechanisms. This suggests that Dienogest might be particularly effective for scattered endometrial lesions. Previous comparative studies often relied on subjective pain assessment to evaluate effectiveness, lacking objective measures such as lesion size reduction. Hence, it is necessary to incorporate additional treatment and evaluation methods into clinical comparative studies of these treatments to elucidate their specific mechanisms of action further.

## Ethical approval

Not applicable.

## Consent

Written informed consent was obtained from the patient for publication of this case report and accompanying images. A copy of the written consent is available for review by the Editor-in-Chief of this journal on request.

## Source of funding

This work was supported by the National High Level Hospital Clinical Research Funding (2022-NHLHCRF-PY-02).

## Author contribution

T.H. wrote the original manuscript; H.Y. provided the case and reviewed the manuscript; W.H.W. guided writing and reviewed the manuscript; Y.F. edited the manuscript.

## Conflicts of interest disclosure

The authors declare that they have no financial conflict of interest with regard to the content of this report.

## Guarantor

Takae Hirono.

## Research registration unique identifying number (UIN)

Not applicable.

## Data availability statement

Not applicable.

## Provenance and peer review

Not commissioned, externally peer-reviewed.
